# Bacteria inhabiting spider webs enhance host silk extensibility

**DOI:** 10.1038/s41598-024-61723-x

**Published:** 2024-05-14

**Authors:** Maryia Tsiareshyna, Te-Hsin Wang, Ying-Sheng Lin, Dakota Piorkowski, Sammi Yen-Ting Huang, Yi-Lun Huang, Wei-Ting Chao, Yuan Jay Chang, Chen-Pan Liao, Pi-Han Wang, I-Min Tso

**Affiliations:** 1 Department of Life Science, Tunghai University, Taichung, Taiwan; 2 Department of Chemistry, Tunghai University, Taichung, Taiwan; 3 Taiwan International Graduate Program, Academia Sinica, Taipei, Taiwan; 4 Department of Biology, National Museum of Natural Science, Taichung, Taiwan; 5 Center for Ecology and Environment, Tunghai University, Taichung, Taiwan

**Keywords:** Major ampullate silk, *Triconephila clavata*, *Microbacterium*, *Novosphingobium*, Exopolysaccharide, Developmental biology, Ecology, Microbiology, Systems biology, Zoology

## Abstract

Spider silk is a promising material with great potential in biomedical applications due to its incredible mechanical properties and resistance to degradation of commercially available bacterial strains. However, little is known about the bacterial communities that may inhabit spider webs and how these microorganisms interact with spider silk. In this study, we exposed two exopolysaccharide-secreting bacteria, isolated from webs of an orb spider, to major ampullate (MA) silk from host spiders. The naturally occurring lipid and glycoprotein surface layers of MA silk were experimentally removed to further probe the interaction between bacteria and silk. Extensibility of major ampullate silk produced by *Triconephila clavata* that was exposed to either *Microbacterium* sp. or *Novosphigobium* sp. was significantly higher than that of silk that was not exposed to bacteria (differed by 58.7%). This strain-enhancing effect was not observed when the lipid and glycoprotein surface layers of MA silks were removed. The presence of exopolysaccharides was detected through NMR from MA silks exposed to these two bacteria but not from those without exposure. Here we report for the first time that exopolysaccharide-secreting bacteria inhabiting spider webs can enhance extensibility of host MA silks and silk surface layers play a vital role in mediating such effects.

## Introduction

Due to their incredible toughness and extensibility, spider silk has great potential in various biomedical applications^1–4^. Silk has unusual physical and chemical properties that are different from any other known natural and synthetic fibers. Alanine-rich crystalline regions are responsible for tensile strength, while glycine-containing motifs are responsible for high extensibility of spider silk^5^. Mechanical properties such as initial modulus and breaking strain, are linked to the ability of fibers to supercontract and both are dependent on variability in the hydrogen-bonded amorphous oriented regions of the silk^6^. Amorphous region is regarded to contribute to absorption and dissipation of energy during the torsion, while β-sheet crystals may maintain permanent shape due to high mechanical stiffness^7^. In addition, spider silk does not trigger an immune response in human cells, making it an ideal material for therapeutic operations^8^. Natural silks are suitable biomaterials for skin regeneration because successful adhesion and proliferation of keratinocytes and fibroblasts has been observed on spider silk woven on frames^9^. Spider silk has been reported to support the growth of chondrocytes and articular cartilage cells, and they can be made into porous scaffolds to facilitate cartilage regeneration^2^. Spider silk also works well as an alignment material for nerve regeneration^10^. Schwann cells migrate and proliferate on spider silk fibers^11^ and axons grow, align and myelinate on natural spider silks^12^. Surgical sutures made from natural spider silks had been shown to exhibit better tensile properties than conventional ones, and therefore could be applied in flexor tendon repair^13^. Spider dragline silks might also be applied in tissue engineering such as bladder reconstruction. Primary human urothelial cells were shown to survive, adhere, grow and even stretch along dragline silks while no cytotoxicity was detected^14^.

One unique feature that makes spider silk a popular material in the aforementioned biomedical applications is its ability to inhibit growth and resist degradation by bacteria^15^. Currently, little is known about the nature and underlying mechanisms of the interactions between spider silk and bacteria. Most studies on the interactions between spider silk and bacteria focus on how the former resisted degradation by the latter and often used commercially available bacteria that have no ecological/evolutionary relationship with natural spider silks^16–20^. Silk collected from webs spun by the house spider *Tegenaria domestica* was shown to be able to inhibit growth of *Bacilus subtitis* bacteria^16^, indicating the presence of an antibacterial substance on spider silks. In another study, three solvents were used to extract substances from webs spun by *T. domestica* spiders and the effects on a series of bacteria were evaluated. The results showed that web acetone extracts inhibited both gram positive and negative bacteria^17^. Acetone extracts from webs spun by the cell spider *Pholcus phalangiodes* were also reported to exhibit antibacterial properties^18^. On the other hand, Zhang et al. (2019) used two bacteria isolated from webs of three species of spiders and found that silks of these spiders did not show antibacterial properties and no inhibition zone was observed around silks^19^. Fruergaard et al. studied antimicrobial activities of spider silk from 7 different species using standard bacterial species *E. coli, B. subtilis and P. putida*. Results revealed no growth inhibition of tested bacteria^20^. However, bacteria could grow on silks of these spiders when extra nutrients were supplemented indicating that spider silk exhibited bacteriostatic rather than bactericidal properties. Currently, microorganism communities inhabiting spider webs are poorly understood and how they influence spider silk is unclear. Bacteria exhibiting a long-term ecological relationship with spider webs may demonstrate novel modes of interaction.

Recent studies have shown that surface layers of natural spider silks play important roles in various interaction between spiders and other organisms, but how surface layers of spider silk are involved in interactions between silks and bacteria inhabiting spider webs is unknown. The surface of spider silk is composed of lipid layer, glycoprotein layer and protein skin layer^20^. The outermost layer is composed of lipids and in some spiders this layer carries pigments, pheromones and hormones involved in intra- and interspecific behavioral interactions^21–23^. In addition to functioning as a visual prey attractant^24^, pigments in the lipid layer contain xanthurenic acid, which has been reported to have antibacterial properties^25^. The glycoprotein layer is composed of spidroin-related molecules and might be involved in protection and water balance^20^. The skin layer is composed of MiSp-like proteins, and might protect dragline silk against protease digestion^26^. Some bacteria were reported to secret certain chemicals to facilitate interactions with the surface of hosts. For example, exopolysaccharides (EPS) are structurally diverse polymers synthesized and secreted by certain bacteria^27^. Members of the genus *Novosphingobium* were reported to be able to produce sphingan, which was characterized by high viscosity and could work as a cement^28,29^. Additionally, members of the genus *Microbacterium* have been reported to produce levan, which exhibits high shear stress and low viscosity^30,31^ and could potentially enable bacteria to adhere to host substrates^32^. Some EPS were shown to be involved in the symbiotic interactions between bacteria and plants. In some bacteria, EPS plays essential roles in enabling the bacteria to adhere to the roots of host plants^33,34^. Moreover, some EPS has been shown to have material mechanical property enhancing effects. For example, adding EPS from *Gluconacetobacter xylinus* to composites with bacterial cellulose could enhance mechanical properties while preserving crystallinity^35^. Therefore, although there is still no relevant literature, EPS can potentially play roles in the interactions between bacteria and silk surface layers.

Currently, whether bacterial EPS is produced on natural spider silk or can enhance tensile properties is unknown. From a preliminary survey of bacterial communities inhabiting spider webs, we identified bacteria that can potentially secrete EPS from the genera *Microbacterium* and *Novosphigobium*. In this study, we isolated such bacteria from webs of the golden orb spider *Triconephila clavata* to evaluate their effects on mechanical properties of major ampullate (MA) silks produced by their hosts. In addition, we also manipulated surface layers of *T. clavata* MA silks to determine their roles in the interaction between bacteria and spider silks. We found that these two bacteria enhanced the extensibility of MA silks produced by *T. clavata* through deposition of EPS, and silk surface layers played a vital role in mediating these effects.

## Methods and materials

### Spider and silk collection

Golden orb spiders *Trichonephila clavata* (Koch, 1878) of the family Nephilidae are widely distributed in low and mid-elevation mountainous areas in Taiwan. While most members of the family Nephilidae are solitary, *T. clavata* aggregate together using communal scaffold silks and deposit lots of prey remains on webs^36^. Spiders were collected from Wushiken, Taichung City, Taiwan and were individually housed in enclosures at Tunghai University. We followed the method reported in Blamires et al. to collect bundles of MA silks (5 mg each sample) directly from large females through forcible extraction using an electric rotor with a reeling rate of 0.01 m/s. Bundles of MA silk samples were later used for transmission electron microscopy (TEM) and nuclear magnetic resonance analyses (NMR)^37^.

### Isolation and culture of bacteria from spiders and webs

Bacterial strains were isolated and identified from the webs of *T. clavata*. Webs including prey remains were collected from the field and were placed into sterile plastic ziplock bags and later liquefied followed by vortexing before being spread on sterile agar plates. Agar plates receiving the aforementioned treatments were then subjected to standard bacterial isolation and culture processes. For each colony of bacteria 16S ribosomal DNA was amplified, sequenced using PCR, and compared to known sequences using BLAST. A total of 22 strains of bacteria were isolated and among them *Microbacterium* sp. (Max. score = 2704, identity = 100%, Accession number JQ793425.1) and *Novosphigobium* sp. (Max. score = 2590, identity = 97.3%, Accession number KF465976.1) were discovered through BLAST identification. These two bacteria were suspended in 5 ml of full nutrient broth and incubated for 24 h at 27 °C. We then added 20% glycerol to the tubes, and stored them at − 80 °C. The bacterial strains in this study were deposited in the Microbial Ecology Laboratory, Department of Life Science, Tunghai University, Taiwan.

### Visualization of *T. clavata* MA silk surface layers

We used transmission electron microscopy (TEM) to visualize various surface layers of MA silks produced by *T. clavata*. MA silk sample was fixed in Karnovsky solution at 4 °C and washed with 0.1 M Cacodylate buffer 10 min thrice and postfixed in 1% OsO4 for 30 min at room temperature. After being postfixed, the pieces were washed thrice in Cacodylate buffer for 10 min. The samples were dehydrated in 50% EtOH for 10 min, moved to a new Eppendorf, and after centrifugation the supernatant was removed. Samples were dehydrated with 75% EtOH (10 min), 95% EtOH (10 min), and then 100% EtOH (10 min for three times). Samples were then transferred to 1,2-propylene oxide/EtOH mixture (1:3, 1:1, 3:1) for 15 min. Subsequently the samples were rinsed with 100% propylene for ten minutes trice and placed in Epon/propylene oxide mixture (1:3, 1:1, 3:1) for 1 h, 4 h, and 8 h and then 100% Epon for 12 h. Finally, samples were embedded in a fresh Eppendorf with fresh Epon at 40 °C for 18 h and 60 °C for 24 h. Samples were cut by using ultramicrotome (Leica Ultracut UC7, Wetzlar, Germany), and thin sections (80 nm) were stained with uranyl acetate and lead citrate and viewed under a transmission electron microscope (Hitachi HT-7700, Tokyo, Japan).

### Manipulation of *T. clavata* MA silk surface layers

We followed the protocols reported by Sponner et al. and Yazawa et al. to remove lipid and glycoprotein layers of MA silks^20,26^. To remove lipid layer, about 5 mg of MA silk samples was submerged in diethyl ether and shaken gently for 10 min. After such operation, the silk samples were rinsed in distilled water then the diethyl ether treatment was repeated again. To remove glycoprotein layer, silk samples receiving lipid removal treatment was placed in Eppendorf containing 0.1% Triton-X-100 and vortexed vigorously for 10 min and such process was repeated for 10 times. Silk samples with lipid and glycoprotein layers removed were examined by NMR to verify the effectiveness of treatments. Treated silk samples were placed in Eppendorf tubes and were added 1 mL of 1,1,1,3,3,3-Hexafluoro-2-propanol-d_2_. Subsequently, 0.5 ml of the resulting solution was extracted from the tube and used for measurement and analysis. The ^1^H NMR spectra of dissolved silk bundles were recorded using a Bruker AVIII HD 400 spectrometer (Bruker Taiwan Co., Hsinchu, Taiwan). Chemical shifts were reported in ppm downfield from (CH_3_)_4_Si, and coupling constants (*J*) were given in Hertz. The NMR spectra of silk samples with intact surface layers and those receiving surface layer treatments were compared to see whether the signals associated with silk surface layers were different between them.

### Experimental design of silk tensile testing

In this study, we used a multi-factorial design to comprehensively evaluate the effects of bacterial species, silk surface layer and bacterial cultural broth on tensile properties of *T. clavata* MA silks (Supplementary Fig. 1). Single thread MA silk samples collected from *T. clavata* were exposed to either *Microbacterium* sp. or *Novosphingobium* sp. and mechanical performances of MA silk threads incubated with or without bacteria were compared. Single strand MA silk samples to be exposed to various bacteria were subjected to three surface layer treatments (layers intact, lipid layer removed and glycoprotein removed). Zhang et al. showed that most bacteria would not proliferate unless extra nitrogen or carbon nutrients were supplemented^19^, so bacteria to be interacted with MA silk samples were cultured in three different broth (no nutrient; minimal nutrient with nitrogen source, without carbon source; minimal nutrient with carbon source, without nitrogen source). We collected 10 mature *T. clavata* females from the field. From each female spider we collected at least 36 single strand MA silk samples to make sure that they were comprehensively used in all factorial treatments (two bacterial species × two treatments × three surface layer manipulations × three broth types) to minimize the confounding effect that silk samples used in various treatments were collected from different individuals.

### Preparation of silk samples for tensile testing

Each female *T. clavata* spider was placed ventral side up unto a Styrofoam block and was immobilized with non-adhesive tapes and pins. Single strand of MA silk was reeled from spider's anterior spinneret at a speed of 0.01 m/s using a rotor powered by an electric motor. The MA silk strand was collected onto a cardboard with a 10 mm × 10 mm gap. Double sided sticky tape was used to affix the silk thread at each end of the cardboard and PVA waterproof glue (Huayu Enterprise Co., Taipei, Taiwan) was applied latter near the edges of gaps. When the glue dried, we mounted the cardboard on microscope slides and divided silk samples into three groups. In the first group, the surface layers of MA silks were kept intact. In the second group the lipid layer of MA silk samples was removed while in the third group the glycoprotein layer was removed. We removed lipid layer of MA silks mounted on cardboards by dropping diethyl ether directly on the silk thread. After it dried out the silk samples were washed by dripping water. The aforementioned procedures were repeated two times and the silk samples were left to dry for 5 min. To remove glycoprotein layer, on half of the silk samples treated with diethyl ether we dripped 0.1% Triton X-100 solution diluted with PBS broth, followed by washing with water. After 15 min the aforementioned processes were repeated again for 10 times.

### Interaction of MA silk and bacteria

We evaluated the effects of *Microbacterium* sp. and *Novosphingobium* sp. isolated from webs of *T. clavata* on tensile properties of host MA silks. Zhang et al. showed that most bacteria would not proliferate unless extra nitrogen or carbon nutrients were supplemented^19^. Therefore, we cultured these two bacteria in three types of broth. In the first one, the PBS broth (phosphate buffer saline, 10 ml 0.5 M KH_2_PO_4_ and 2.5 mL 0.4 M MgCl_2_·6H_2_O, with 2 L water), no extra nutrient was supplemented. In the second one, the NFG broth (minimal nutrient without nitrogen source, 13.6 g KH_2_PO_4_, 7.1 g NaHPO_4_, 0.25 g MgSO_4_·7H_2_O, 0.01 g CaCl_2_ and 8 g Glucose, with 1 L water), extra carbon was supplemented^16^. In the third one, the CFN broth (minimal nutrient without carbon source, 13.6 g KH_2_PO_4_, 7.1 g NaHPO_4_, 0.25 g MgSO_4_·7H_2_O, 0.01 g CaCl_2_ and 5 g (NH_4_)^2^SO_4_), extra nitrogen was supplemented. For each bacterium species, 5 μL of bacterial suspension (105–106 cells/mL) was dripped onto the center part of the silk thread mounted on the cardboard, then the silks were incubated at 28 °C for 24 h. Broth without bacteria was also applied to the silk samples following the same procedures as the control. All operations were carried out under sterile conditions to avoid contamination. The bacteria used in this present study were deposited in Microbial Ecology Laboratory, Tunghai University, Taiwan.

### Testing of silk tensile properties

After being subjected to bacterial exposure and incubation, samples were air dried from extra humidity and broth to exclude its effect on extensibility. Each cardboard with the mounted silk sample was taped to a glass slide and photographed at 100 × using a digital camera (Canon EOS 650D, Tokyo, Japan) attached to a polarized light microscope (Olympus BX53, Tokyo, Japan). We measured diameter of each silk samples using *Image J* program^38^. Then, we performed mechanical tests for each 10 mm silk sample (within two weeks after the completion of treatments) using a Nano Bionix tensile tester (MTS Systems Corp., Eden Prairie, MN, USA) at the Center for Measurement Standards, Industrial Technology Research Institute, Hsinchu, Taiwan. Silks were stretched at a rate of 1% gauge length per second until rupture to generate the load-extension data. All testing was conducted under controlled ambient temperature and humidity (20℃, 30% RH). Young’s modulus, maximum stress, extensibility (i.e., breaking strain) and toughness were derived from stress–strain curves plotted by the program Test Works 4.0 as described by Piorkowski et al.^39^.

### Detecting presence of bacterial exopolysaccharide

First, spider silk was reeled manually onto a glass ring to create a web mesh and a total of three such rings were prepared. In the first two silk sings, a few droplets (with a total volume of 5 µL) of either *Microbacterium* sp. or *Novosphingobium* sp. cultured in NFG broth were dripped by pipette onto spider silk mesh. In the third ring same amount of NFG broth was dripped on silk mesh to serve as a control. All three rings were then incubated at 28 °C for 24 h. After incubation, silk form the rings was collected, placed into Eppendorf, weighted, and proceeded to NMR operation. The ^1^H NMR spectra were recorded using a Bruker AVIII HD 400 spectrometer. Chemical shifts were reported in ppm downfield from (CH_3_)_4_Si, and coupling constants (*J*) were given in Hertz. Spider silk samples (20 mg each) was mixed with D_2_O and soaked in an Eppendorf tube for 15 min. Subsequently, 0.5 mL of the resulting D_2_O solution was extracted from the tube and used for measurement and analysis.

### Statistical analyses

We conducted multivariate analysis to determine the explanatory variable structure and then conducted univariate analyses for each tensile properties according to the same structure. We performed partial redundancy analysis (pRDA) to compare the tensile properties (i.e. maximum strain/stress, toughness and modulus) of MA silk among treatments. Method pRDA, as a multivariate analysis technique, provides the capability to simultaneously model silk properties, assess linear effects and/or interactions, and account for the random effect of spider identity, which is not the primary focus of this study. We first calculated the averages of each tensile properties obtained from same spider under same treatment to avoid pseudo-replication issue, and then we natural-log transformed all averaged tensile properties to cope with heteroscedasticity. Four tensile properties were assigned as the response variables. The following four explanatory factors were included: bacteria treatment (with/without bacteria), layer type (G/L/N), bacterial species (*Microbacterium* vs. *Novosphingobium*) and broth type (CFN/NFG/PBS). Interaction(s) among main factors was included when permutation P (Pperm) was less than 0.1 during a forward stepwise process with permutation test, which shuffled the pRDA residuals within each spider individual. Spider individual identity was also included as the conditioning factor to achieve the comparisons within individual. After variable structure was determined, we performed linear mixed models (LMM) to fit each tensile property with the same RHS structure. To cope with the pseudo-replication issue and achieve comparisons within spider identity, the following two random factors were assigned: spider individual identity and the treatment within spider individual identity, and the data for LMMs were not averaged beforehand. P-values of each parameter among different tensile properties were gathered and adjusted by using the Benjamini–Hochberg method^40^ to control the false discovery rate (FDR). To present the effect size between treatments, we estimate the Cohan’s *d*,

$${\delta }_{t}=\frac{{\beta }_{t}}{\sqrt{{\sigma }_{{\text{spider}}}^{2}+{\sigma }_{{\text{spider}}:{\text{sample}}}^{2}+{\sigma }_{{\text{residual}}}^{2}}}$$

where β_t_ denotes the expected difference between two treatments, and σ_spider_, σ_spider:sample_ and σ_error_ denote the SD among spider individual identities, the SD among treatments within each spider individuals and the regression residuals, respectively^41^.

All of the pRDAs and LMMs were computed by the R package ‘vegan’ v. 2.6-2^42^ and ‘lmerTest’ v. 3.1-3^43^, respectively. All analyses were conducted under the R environment v. 4.2.0.

### Ethical statement

All experiments were conducted in compliance with the “Research Ethics and Animal Treatment” legal requirements of Tunghai University, Taiwan. For the organisms used and operations conducted in this study no permissions are required.

## Results

### Visualization of MA silk surface layers and verification of treatment effects

**Figure 1:**
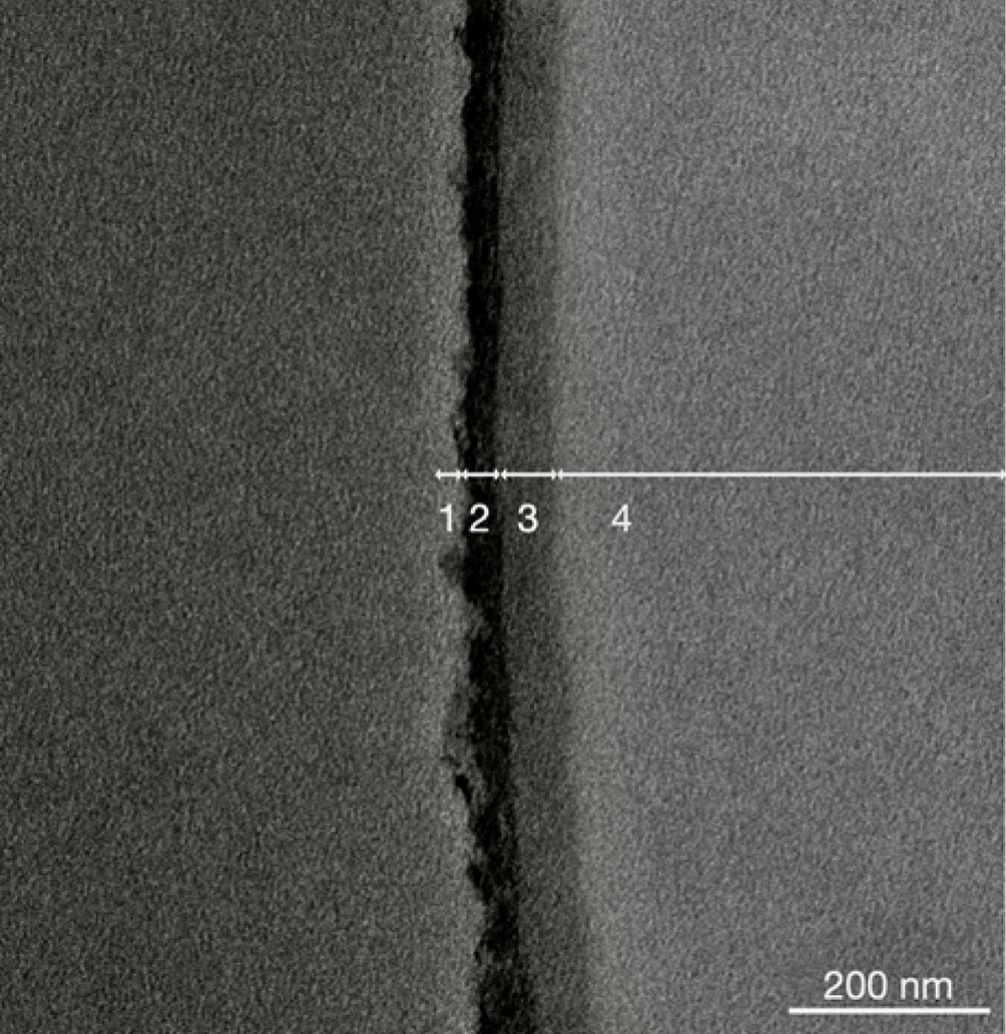
Transmission electron microscopy images of stained layers along the edge of a cross-section of a major ampullate silk fiber of *Trichonephila clavata.* Numbers indicate identity of layers: 1—lipid layer, 2—glycoprotein layer, 3—protein skin layer, 4—bulk protein.

**Figure 2:**
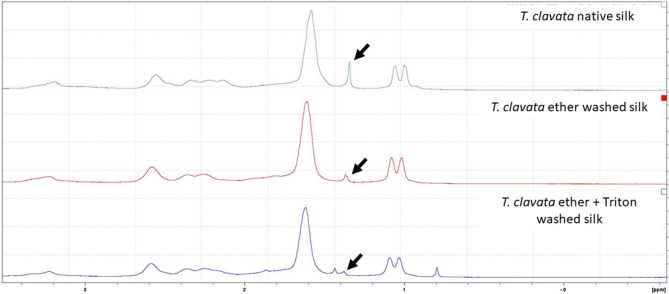
Results of NMR of native, ether washed, and ether plus 0.1% Triton X washed major ampullate silk (MAS) fibers collected from *Triconephila clavata*. Each spectrum was produced using MAS from a single individual spider. Arrows indicate the putative identification of a methyl group of the lipid layer at 1.4 PPM.

The thickness of the lipid layer of *T. clavata* MA silks was around 40–50 nm, while those of glycoprotein and skin layers were 40–50 and 60–80 nm, respectively (Fig. 1). The thickness of various layers was similar to those of *T. clavipes* MA silks reported by Sponner et al.^20^. Removal of the glycoprotein layer through washing with ether and Triton X caused the coloration of MA silk fibers to change from bright yellow to pale white. The effects of layer removal treatments could be reflected in the TEM image (Supplementary Fig. 2) and chemical composition changes. Removal of lipid layers by washing was confirmed by NMR (Fig. 2), as the putative methyl group signature of lipids was greatly reduced after diethyl ether and ether + 0.1% Triton X 100 washes.

### Effect of two bacteria on MA silk tensile properties

**Figure 3:**
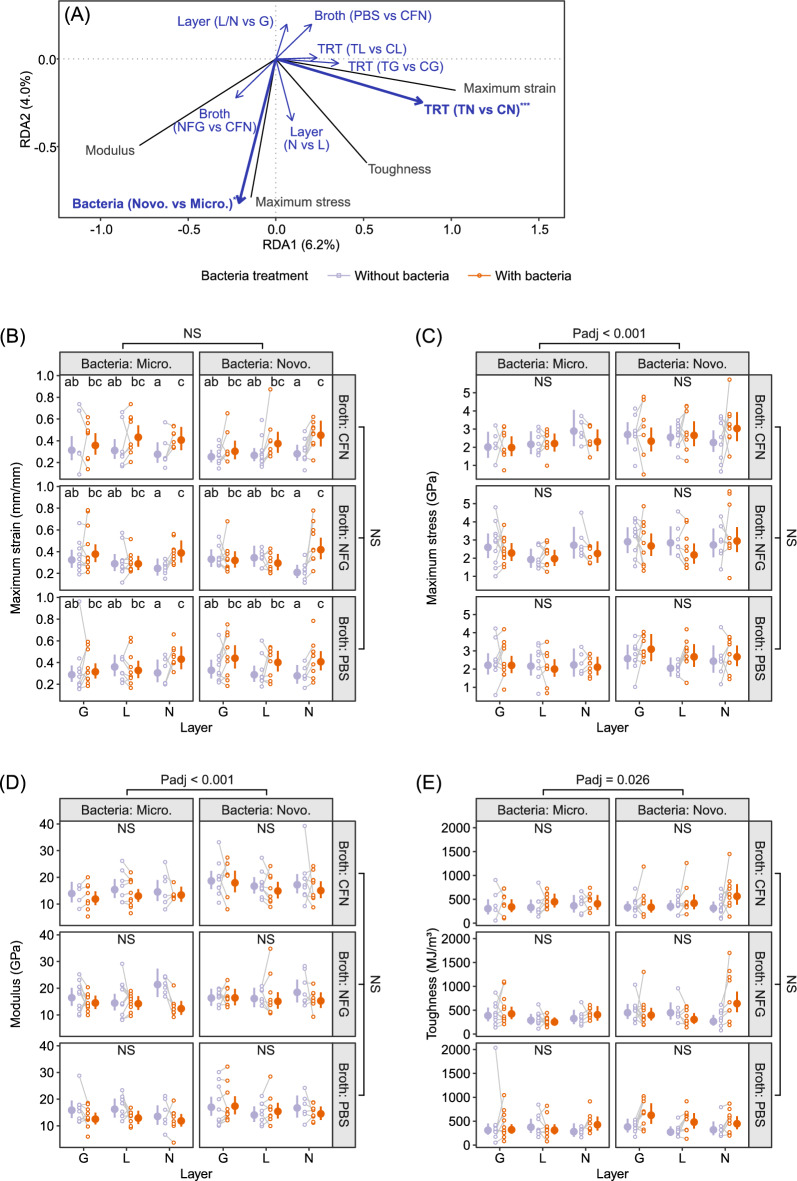
Ordination plots of the best partial RDA models fitting tensile properties (**A**) and the univariate models fitting maximum strain (**B**), maximum stress (**C**), modulus (**D**) and toughness (**E**) of *T. clavata* MA silks. The red lines and the black lines in panel A represent the projections of explanatory factors and response variables, respectively. Bold blue texts and thicker blue lines indicate the significant explanatory factors. Lowercase letters in panel (**B**–**E**) indicate the summary of multiple comparisons among layer-treatment groups. *TRT* bacteria treatment, *TL* receiving both lipid removal and bacterial treatment, *CL* receiving lipid removal but no bacterial treatment, *TG* receiving both glycoprotein removal and bacterial treatment, *CG* receiving glycoprotein removal but no bacterial treatment, *TN* receiving bacterial treatment but no surface layer removal, *CN* receiving neither bacterial treatment nor surface layer removal, *NS* not significant, *Padj* FDR-adjusted p-value, ***P < 0.0001. *Layer* surface layer treatment, *L* lipid layer removal treatment, *G* glycoprotein removal treatment, *N* receiving no layer removal treatments, *Micro Microbacterium* sp., *Novo Novosphingobium* sp.

The outcome of the pRDA model revealed that various tensile properties of MA silks exposed to two bacteria differed and the interaction between layer removal and bacteria exposure was significant, which marginally contributed 2.3% and 4.8% adjusted- R^2^, respectively (Fig. 3A, Table 1). MA silk samples exposing to different bacteria differed significantly in toughness. Compared to MA silks exposing to *Microbacterium* sp., those exposing to *Novosphigobium* sp. were 19.2% (95% CI [10.0%, 29.1%]) higher in maximum stress (δ = 0.41, 95% CI [0.22, 0.60]; Fig. 3C), 14.9% (95% CI [7.7% to, 22.6%]) higher in modulus (Hedges’ g = 0.41, 95% CI [0.22, 0.61]; Fig. 3D) and 15.6% (95% CI [2.5%, 30.2%]) higher in toughness (δ = 0.24, 95% CI [0.04, 0.44]; Fig. 3E) (Fig. 3C–E). Silk surface layers seemed to play an essential role in the interaction between two bacteria and MA silks. For MA silk samples with intact surface layers, the maximum strain of those exposing to bacteria was significantly higher than those without (58.7%, 95% CI 26.8% to 98.6%) (δ = 1.09, 95% CI [0.56, 1.61]; Fig. 3B). However, for MA silk samples with either lipid or glycoprotein removed, no such effects were detected (lipid removed, 15.1% higher, 95% CI [− 5.9%, 41.0%], δ = 0.33, 95% CI [− 0.14, 0.81]; glycoprotein removed, 16.1% higher, 95% CI [− 5.1%, 41.9%], Hedges’ g = 0.35, 95% CI [− 0.12, 0.82]; Fig. 3B). The type of broth used in mediating silk-bacterial interactions did not have significant effects on silk tensile properties (Table 1).

**Table 1:** The results of multivariate variation partitions, significance tests and univariate significance test according to the multivariate model after variable selection.

Independent variable	Marginal adj-R^2^	Marginal Pperm	FDR adjusted p-value of univariate analysis			
			Max strain	Max stress	Modulus	Toughness
Layer	–	–	0.9537	0.3553	0.5313	0.5313
Treatment	–	–	**0.0004**	0.9856	**0.0004**	**0.0009**
Layer × treatment	0.0488^†^	**0.0377**	**0.0128**	0.8583	0.3554	0.0714
Broth	− 0.0004	0.3512	0.6358	0.9565	0.6358	0.9773
Bacteria	0.0230	**0.0001**	0.7763	**0.0002**	**0.0002**	**0.0258**

^†^Including the effects from layer, treatment and their interaction.

### Presence of bacterial EPS revealed by NMR

**Figure 4:**
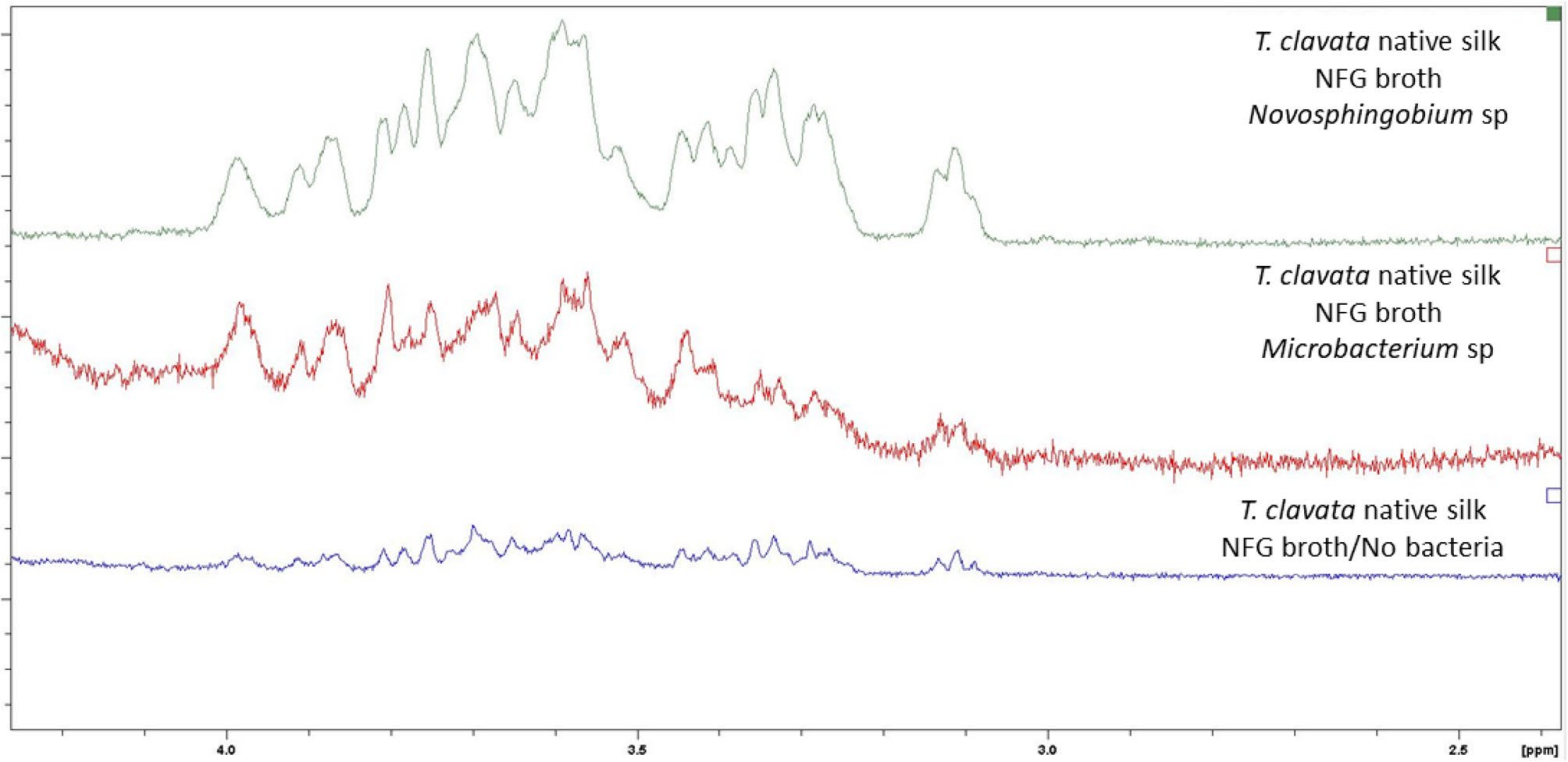
The 1D ^1^H spectra (OCH and OCH_2_ units recorded at 500 MHz in D_2_O at 50 °C) of *Trichonephila clavata* MA silk samples incubated with either *Novosphingobium* sp. or *Microbacterium* sp. as well as that receiving control treatment, indicating presence of bacterial EPS in MS silk exposed to bacteria.

Results of NMR analyses showed that EPS signals were detected from *T. clavata* MA silk samples exposed to either *Novosphingobium* sp. or *Microbacterium* sp. cultured in NFG broth. The 1D ^1^H spectra of MA silk samples exposing to *Novosphingobium* sp. and *Microbacterium* sp. as well as that receiving control treatment were shown in Fig. 4. From these spectra we focused on signals of carbinolic protons at 3.0 to 4.0 ppm because previous studies demonstrated that peaks at such interval represented those of bacterial EPS^44,45,46^. The signal peaks for *Leuconostoc citricula* E497 appeared between 3.4 ppm and 3.98 ppm in proton NMR, which suggested that the structure may contain spider web-like polysaccharides. It is speculated that this structure may be the most probable polysaccharide species within the 3–4 ppm range. In this study, no hemi-acetal signal (5.2 ~ 5.4 ppm) was observed in the carbohydrate structure, which was attributed shielding by ring flip of carbohydrate structure. For MA silk samples incubated with either *Novosphingobium* sp. or *Microbacterium* sp. high carbinolic proton signal intensity was observed in such interval. However, the intensity of corresponding signals of MA silk samples receiving control treatment was low. The distribution pattern of ^1^H NMR signal peaks in spectra derived from MA silk incubated with two species of bacteria seemed to be quite similar. Between the carbohydrate-related NMR resonances of MA silk samples incubated with different bacterial species only rather minor variations (OCH and OCH_2_ units) were found, indicating presence of very similar EPS carbohydrates in two web-inhabiting bacteria.

## Discussion

Our study showed that EPS-secreting bacteria inhabiting webs can enhance material properties of spider silk. Most studies on the interactions between spider silk and bacteria focus on how the former resisted degradation by the latter and often used commercially available bacteria that have no ecological/evolutionary relationship with natural spider silks. Currently, microorganism communities inhabiting spider webs are poorly understood and how they influence spider silk is unclear. Zhang et al. used two species of bacteria isolated from webs of three species of spiders instead of commercial strains and found that silks of these spiders did not show antibacterial properties. However, in that study whether the presence of bacteria naturally inhabiting spider webs would affect silk properties was not examined. In this present study, by subjecting bacteria exhibiting a long-term ecological relationship with spider webs to interact with spider silks, we demonstrate for the first time a novel mode of interaction between spider silks and bacteria^19^. Members of the bacterium genera *Microbacterium* and *Novosphingobium* were identified and isolated from the natural microbial communities inhabiting webs of *T. clavata*. Members of the genus *Novosphingobium* are gram negative soil-dwelling bacteria capable of degrading compounds such as phenol, aniline, nitrobenzene and cellulose^47,48^. Members of *Microbacterium* are gram positive bacteria found in soils, insect guts and plant leaves and may exhibit antifungal activities^49^. When these two web-inhabiting bacteria were used to interact with spider silks, we discovered a novel relationship between spider silks and bacteria. *T. clavata* spiders tend to aggregate together using long-lasting communal silk scaffoldings and deposit prey remains on webs^36^. Such behaviors create a favorable microenvironment for microbial communities inhabiting spider webs. The prey carcasses deposited by *T. clavata* on webs might provide nutrients for inhabiting bacterial communities and the long-lasting communal scaffolds might serve as a stable habitat for proliferation of bacteria. Results of our study showed that *T. clavata* and these two bacteria seemed to form a symbiotic relationship. The host spiders provide nutrients and stable microhabitats for bacteria, while bacteria secrete EPS which enhance silk extensibility and consequently increase durability and wind resistance of *T. clavata* web complexes. In a preliminary study we also subjected MA silks extracted from *Nephila pilipes* (a solitary spider which did not deposit prey on webs and rebuilt their webs on a daily basis) to interact with *Microbacterium* and *Novosphingobium* but did not detect the strain-enhancing effect observed in this present study (I. M. Tso. Unpublished data). Such result indicates that the ecological relationship between bacteria and spider silks is very diverse and complicated and more efforts are needed to expand our knowledge of interactions between spider silks and co-inhabiting bacteria.

We found that members of *Microbacterium* and *Novosphingobium* isolated from webs of *T. clavata* enhanced maximum strain of such spiders' MA silks. Some members of the genus *Novosphingobium* were reported to be able to produce EPS characterized by high viscosity^25,26^ and researchers suggested that polysaccharide polymers produced by such bacteria could potentially improve fiber extensibility^50–52^. Some members of the genus *Microbacterium* had been reported to produce EPS with high shear stress and low viscosity^31,32^ and such a polymer could potentially enable bacteria to adhere to substrates, such as spider silk surfaces^30^. Most EPS are characterized by high viscosity and thermal/pH stability, and some were shown to exhibit biofilm-forming abilities and gelling capacities^31^. Opell et al. demonstrated that spider radial silk fibers coated with glue droplets became viscoelastic and more extensible compared to untreated fibers^53^. Such a mechanism might explain why the presence of EPS-secreting bacteria can enhance the maximum strain of spider MA silks. The EPS produced by these two web-inhabiting bacteria might exhibit properties similar to that of sticky droplets and consequently rendered the MA silks more extensible.

Spider silks have long been known to be a promising biomaterial for industrial and biomedical applications due to their exceptional mechanical properties. However, under certain circumstances some tensile property modifications are needed to render the silks suitable for specific purposes. One of the main advantages of spider silk is that while being a though material it is also highly extensible^54^. Here we show for the first time that silk extensibility can be improved naturally by EPS-secreting bacteria inhabiting spider webs. Researchers working on creating biomaterials for wound healing might be benefited from such finding. For example, collagen gel constructs cultured with elastogenic factors (EFs) stretched at 1.5 Hz has shown maximum elastin mRNA expression and total matrix elastin^55^. Magnetic stretching was applied on human periodontal ligament stem cells (hPDLSCs) within an aligned rat collagen scaffold. Results showed evidence for ECM remodeling because stretching enhanced initial ECM degradation and new ECM secretion, which processes were vital for connective tissue development^56^. Knowledge about how EPS-secreting bacteria influence material elasticity will contribute to improvement of proliferation and adhesion of cells, which properties are critical for tissue engineering.

Currently, microbial communities on spider webs do not receive much attention. In our study, from microbial communities inhabiting webs of *T. clavata* spiders we discovered two EPS-secreting bacteria that can enhance host silk extensibility. Such results indicate that there might be a high diversity of EPS-producing bacterial fauna inhabiting spider webs capable of producing a huge array of polysaccharide polymers unknown to science but with enormous application potential. Recently, the structurally diverse bacterial EPS has been used in various biomedical applications. EPS can serve as microsphere vectors for drug delivery and has been shown to be capable of promoting intestinal health, altering microorganism composition, enhancing immune activity, and improving blood flow^27,57^. For example, dextran can serve as effective plasma substitute and xanthan gum is used in food, cosmetics, industries and agriculture^27^. Sphingans produced by members of *Novosphingobium* can be used as an agar substitute and are applied in food and pharmaceutical industries^58^. Bacterial EPS can be applied in anti-tumor and anti-inflammatory therapeutic operations^31^. Additionally, EPS are known to be suitable drug delivery agents due to their non-toxicity and biocompatibility^59^. Since spider silks also have great potential for drug delivery^15,60^, EPS might be able to modify and enhance the performance of silk-based drug carriers. More efforts should be given to investigate microbial communities inhabiting webs of various spiders to discover more EPS-producing microorganisms and to explore their biomedical and industrial potential.

Both *Microbacterium* sp. and *Novosphingobium* sp. could increase silk extensibility of MA silks produced by *T. clavata* and such an effect was most noticeable when silk surface layers were intact. We found that when the surface layers of spider MA silks were intact the presence of these two bacteria could increase maximum strain by 60%, but such enhancing effects were not observed when silk surface layers were removed. This result indicated that silk surface layers were involved in interactions between *T. clavata* MA silks and web-inhabiting EPS-secreting bacteria. Surface layers had been demonstrated to play important roles in interactions between spiders and a diversity of arthropods mediated through the pheromones and pigments in these layers^22,23,25^. Results of this present study further demonstrate that surface layers also play essential role in interactions between spiders and EPS-secreting microorganisms. The lipid layer might facilitate EPS production because biosynthesis pathways of certain bacterial EPS involve lipid-linked cycles^61^. On the other hand, the glycoprotein layer might help bacteria to attach to silk surface because glycoproteins are involved in promoting attachment of some bacteria to host plant roots^62^. Future studies on natural bacterial communities inhabiting spider silks and mechanisms of their interactions not only will greatly expand our knowledge form microbial ecological perspectives but will also facilitate many industrial applications. We suggest that more efforts be given to determine the roles various surface layers play in silk-mediated interactions between various spider species and microorganisms and relevant findings will have great biomedical application potential.

### Supplementary Information

Supplementary Figure 1.

Supplementary Figure 2.

## Data Availability

Raw dataset is available under the following link: https://github.com/sacrum60s/Bacteria-silk.git.
