# Supplementary material for: Bacteria inhabiting spider webs enhance host silk extensibility
Source: Sci Rep. 2024 May 14;14:11011. doi: 10.1038/s41598-024-61723-x (PMC11093983; doi:10.1038/s41598-024-61723-x)
Supplement: Supplementary file 1 — Supplementary Figure 1. [file 41598_2024_61723_MOESM1_ESM.pdf]

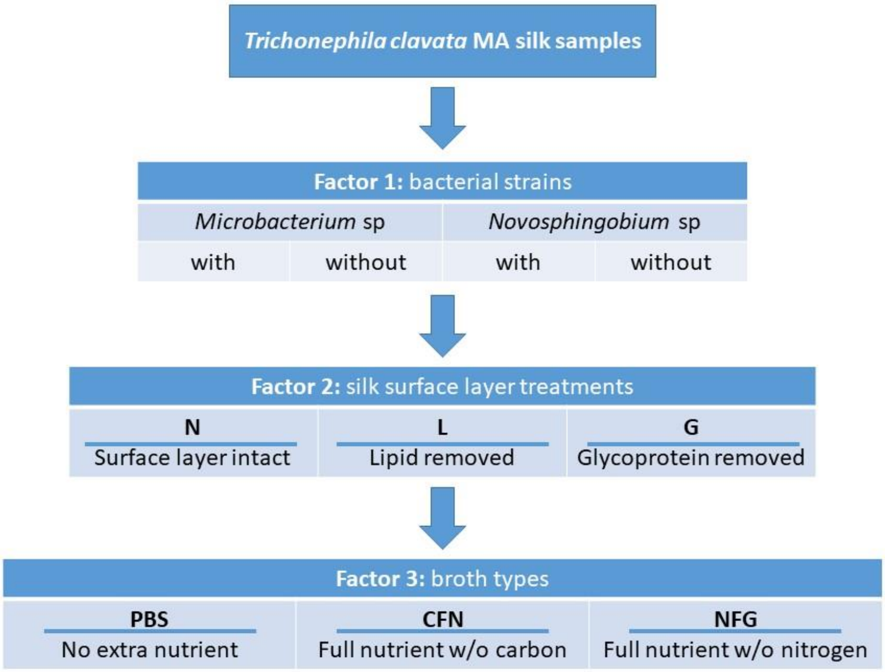

Supplementary Figure 1. A diagram demonstrating the 4×3×3 factorial experimental design of this study. Major ampullate (MA) silks collected from *Triconephila clavata* spiders were interacted with two species of bacteria isolated from webs. MA silk samples to be interacted with bacteria was subjected to three surface layer treatments. Bacteria to be exposed to various silk samples were cultured in three broth types.
