# Supplementary material for: Bacteria inhabiting spider webs enhance host silk extensibility
Source: Sci Rep. 2024 May 14;14:11011. doi: 10.1038/s41598-024-61723-x (PMC11093983; doi:10.1038/s41598-024-61723-x)
Supplement: Supplementary file 2 — Supplementary Figure 2. [file 41598_2024_61723_MOESM2_ESM.pdf]

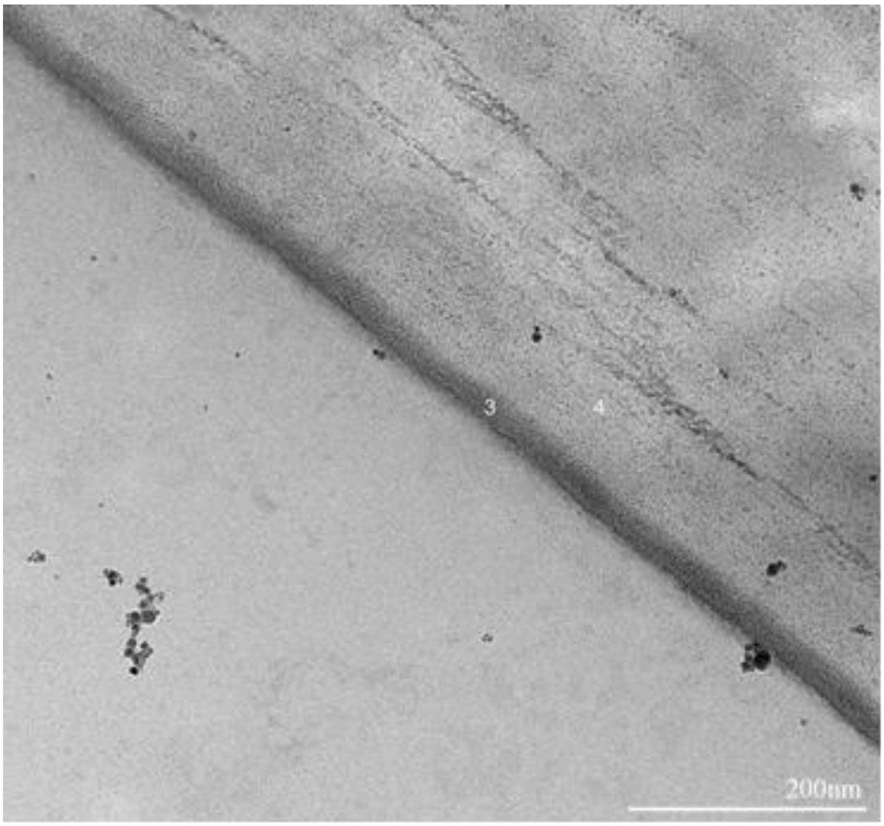

Supplementary Figure 2. Transmission electron microscopy images of stained layers along the edge of a cross-section of a major ampullate silk fiber of *Trichonephila clavate* after removal of lipid and glycoprotein layers. Numbers indicate identity of layer: 3 – protein skin layer, 4 – bulk protein.
